# Non-contrast low-dose CT can be used for volumetry of ADPKD

**DOI:** 10.1186/s12882-023-03359-z

**Published:** 2023-10-26

**Authors:** Jaeyeong Yoo, Jin up Kim, Jisu Kim, Sohyun Jeon, Young-jin Song, Kwang-ho Choi, Seok-hyung Kim, Jong-woo Yoon, Hyunsuk Kim

**Affiliations:** Department of Internal Medicine, Hallym University Medical Center, Chuncheon Sacred Heart Hospital, Chuncheon-si, Gangwon-do 24253 Republic of Korea

**Keywords:** ADPKD, Agreement, Correlation, Ellipsoid method, Stereology, volumetry

## Abstract

**Background:**

Kidney volume provides important information for the diagnosis and prognosis of autosomal dominant polycystic kidney disease (ADPKD), as well as for the evaluation of the effects of drugs such as tolvaptan. Non-contrast computed tomography (CT) is commonly used for volumetry, and this study examined the correspondence and correlation of kidney volume measured by standard-dose or low-dose CT.

**Methods:**

Axial standard-dose and low-dose CT images with 1-mm slices were obtained from 24 ADPKD patients. The kidney was segmented in the Synapse 3D software and the kidney volume was calculated using stereology. The kidney volume was compared between the two sets of images using R^2^, Bland-Altman plots, coefficient of variation, and intra-class correlation coefficients (ICCs).

**Results:**

The mean age of the 24 patients was 48.4 ± 10.9 years, and 45.8% were men (n = 11). The mean total kidney volume on standard-dose CT was 1501 ± 838.2 mL. The R^2^ of volume between standard-dose and low-dose CT was 0.995. In the Bland-Altman plot, except for one case with a large kidney volume, the two measurements were consistent, and the coefficient of variation and ICC were also good (0.02, 0.998). The CT radiation dose (dose-length product) was 229 ± 68 mGy·cm for standard-dose CT and 50 ± 19 mGy·cm for low-dose CT. A comparable volume was obtained with 20% of the radiation dose of standard-dose CT.

**Conclusions:**

Standard-dose and low-dose CT showed comparable kidney volume in ADPKD. Therefore, low-dose CT can substitute for ADPKD volumetry while minimizing radiation exposure.

**Supplementary Information:**

The online version contains supplementary material available at 10.1186/s12882-023-03359-z.

## Background

Autosomal dominant polycystic kidney disease (ADPKD) is the most common life-threatening genetic kidney disease. There are about 12 million ADPKD patients over the world, and 70% of them progress to end-stage renal disease (ESRD) [1]. According to the Mayo classification, typical ADPKD is divided into five classes. Based on measurements of the height-adjusted total kidney volume (htTKV), the estimated yearly kidney growth is calculated in terms of percentages (E: over 6.0%, D 4.5–6%, C: 3–4.5%, B: 1.5–3%, and A: under 1.5%) [2, 3]. It is well known that kidney function decreases rapidly after the TKV increases significantly, which means that the total kidney volume (TKV) is a key predictor of ADPKD patients’ prognosis, since a decline in kidney function can significantly affect patients’ quality of life (QoL) and produce complications [4, 5]. Thus, in previous clinical trials, the researchers measured annual TKV values, which are an important endpoint for ADPKD prognosis [6–8]. Additionally, TKV is the official criterion for whether to approve tolvaptan for the treatment of ADPKD (TKV ≥ 750 mL or TKV growth rate ≥ 5%/year or 6 months) in Japan [9]. When patients are diagnosed with ADPKD, it is usually recommended to measure the TKV for the Mayo classification and to re-measure it in 2–3 years to calculate the progression rate. Subsequent management varies according to the Mayo classification grade; for instance, the estimated glomerular filtration rate (eGFR) is measured to determine whether to administer tolvaptan for Mayo class C, D, or E [10]. Hence, measuring the TKV is important for making an accurate diagnosis, deciding upon the initial management plan, and predicting the prognosis of ADPKD patients [11–13].

Computed tomography (CT) and magnetic resonance imaging (MRI) are the main modalities used to measure TKV in ADPKD patients. Although CT poses a danger due to radiation exposure (e.g., 10–20 mSv for abdominal CT) [14, 15], it still has many advantages over MRI. It only requires a short acquisition time and has relatively universal acquisition protocols [16]. Furthermore, CT images are easy for clinicians to understand. In addition, the ability of CT to acquire detailed images (e.g., with 1-mm slices) in a short time enables more detailed volume measurements. In general, it can be difficult to perform accurate volumetry using MRI because images with 3- to 5-mm slices are obtained due to issues relating to the lower resolution and longer scan time [17]. MRI may require two coils in patients with very large kidneys or liver. Moreover, MRI is more costly and may not be covered by insurance reimbursements [18, 19], and requires rigorous quality control [16]. Additionally, MRI scans sometimes show black boundary artifacts and ambiguous boundaries [20], making it difficult for clinicians to analyze the images. Of course, an advantage of MRI is that it shows good tissue contrast [21], but there are still several critical limitations in using MRI for TKV measurements in ADPKD patients (Table 1).

**Table 1:** Advantages and disadvantages of CT and MRI for ADPKD volumetry

	CT	MRI
Disadvantages	Radiation exposure	Cost / Reimbursement / Sometimes needs 2 coils
Acquisition time	around 1.5 min	around 15 min
Slice thickness	1–3 mm	3–5 mm
Acquisition protocol	Relatively universal	Needs rigorous quality control
Analysis difficulty	Easy for the clinician to understand	Black boundary artifact / Ambiguous boundary

*Abbreviations:* CT, computed tomography; MRI, magnetic resonance imaging; ADPKD, autosomal dominant polycystic kidney disease

There are several methods to measure the TKV: the ellipsoid method, manual planimetry, and stereology. The ellipsoid technique is commonly used to generate a rapid measurement of kidney volume, but it is subject to inaccuracies [22, 23]. Manual planimetry, which is considered the gold standard for measuring TKV [24], involves multiplying each contouring of kidney slices by the slice thickness, which takes about 30 min, substantially longer than would be feasible in a clinical context. Stereology, which could be an alternative to manual planimetry, is based on counting grid points and multiplying them by grid square area and slice thickness. Stereology takes less time—11 min for MRI and 14 min for CT—but it is still time-consuming, making it unrealistic for clinicians to use these methods in clinical settings other than research, even though these methods are quite accurate [25]. Therefore, renal volume measurement programs based on statistical calculations using AI techniques to analyze CT or MRI scans have been actively developed, especially in America and Europe [26–30]. In this context, Synapse 3D software, as it is widely used for imaging studies, could be a new tool for clinicians to easily obtain TKV measurements that fairly closely correspond to the volume measurements made by planimetry or stereology within seconds. Although many automated volumetry programs are based on MRI, Synapse 3D can use CT scans, which enables it to be more widely used, especially in countries where CT is a common modality, such as Japan and South Korea. Due to the more widespread use of CT, developers in Korea have mainly used CT images for AI-based renal volume measurement programs [11].

An annual total kidney volume (TKV) growth rate of > 5% measured by planimetry or stereology has been considered a radiologic biomarker for risk of rapid progression in Japan. Japanese doctors conduct CT or MRI scans of ADPKD patients at intervals of 6 months or 1 year, and decide whether tolvaptan is covered by insurance according to the TKV growth rate [31]. However, a problem with regular follow-up CT scans is the cumulative radiation dose [32, 33]. Recent programs using AI to measure kidney volume have been mostly based on standard-dose CT images [34–36]; thus, the need to use low-dose CT to reduce the cumulative dose has grown. A recent study by Bevilacqua et al. [37] proposed the idea that low-dose CT might result in similar measurement accuracy to MRI. Through analyzing kidney images obtained using low-dose CT and ultra-low-dose CT (with radiation doses 1.4 times and 2.6 times lower than the standard dose of radiation, respectively), they calculated the TKV using three kidney measurement equations (the traditional ellipsoid, the Mayo ellipsoid, and the mid-slice method). The measurement results were consistent with the reference standard (MRI planimetry), demonstrating the possibility that ellipsoid techniques based on low-dose CT could substitute for MRI planimetry, which takes a very long time. However, it remains unclear whether low-dose CT could be used for accurate stereology reconstructions compared to standard-dose CT. Hence, we took a further step to evaluate whether automated TKV computation using stereology—due to the inaccuracy of the ellipsoid technique—could yield accurate measurements even using low-dose CT images in as short of a time as with the ellipsoid method.

The lack of studies demonstrating that low-dose CT could be a reference method of volumetry instead of standard-dose CT limits the free use of low-dose CT for kidney volume measurements in ADPKD patients. Therefore, this study attempted to demonstrate that low-dose CT can be used as an alternative to standard-dose CT for volumetry, which might enhance the utilization of stereology, as a more accurate technique, by clinicians.

## Methods

**Fig. 1:**
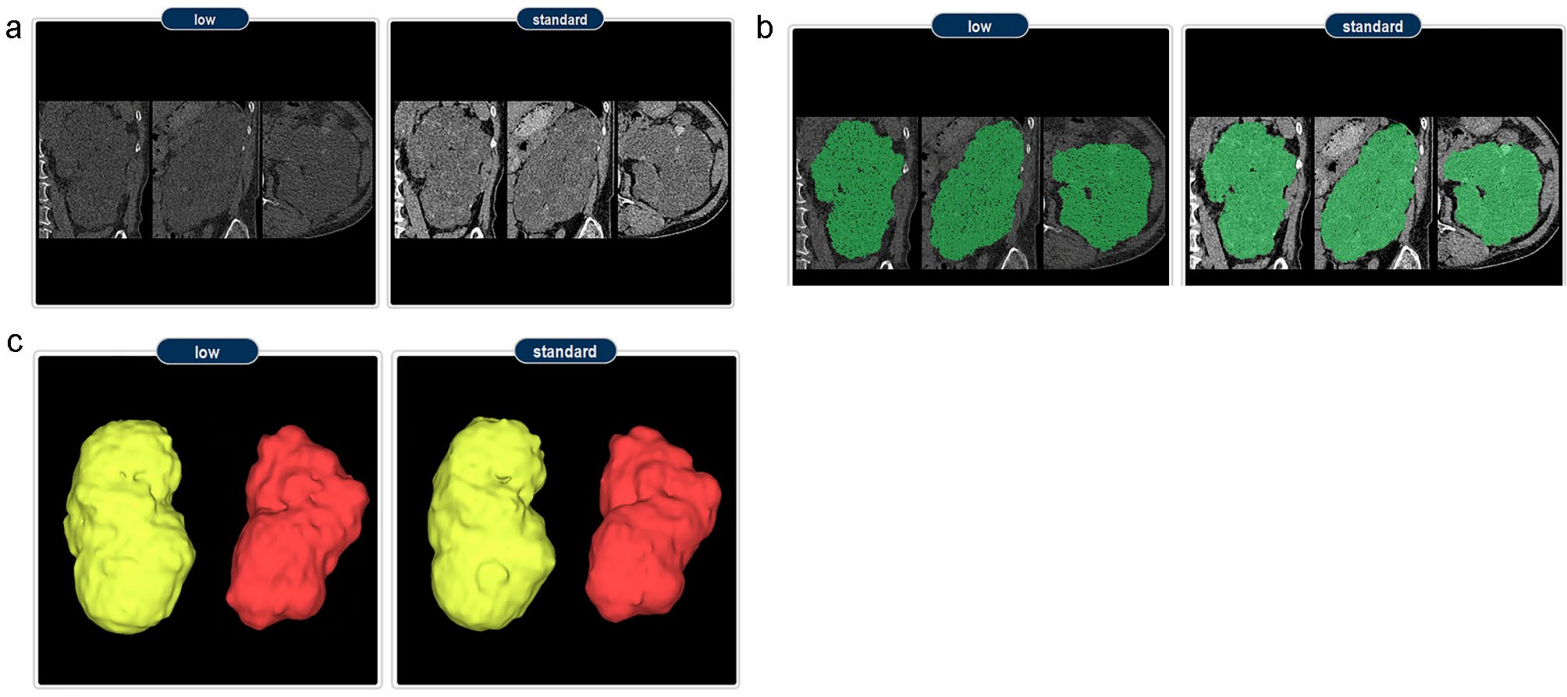
Process of obtaining 3D kidney volume from axial images. (**A**) CT scans from an ADPKD patient are shown (low-dose CT image and standard-dose CT image, respectively.) (**B**) Green areas of the kidney segmentation images are the areas identified by the Synapse 3D software as kidney tissue in ADPKD patients. (**C**) Final 3D reconstructed images of the whole kidneys created by the Synapse 3D software based on consecutive 2D images. 3D, three-dimensional; CT, computed tomography; ADPKD, autosomal dominant polycystic kidney disease; 2D, two-dimensional

Axial standard-dose and low-dose CT images with 1-mm slices were obtained from 24 ADPKD patients after they provided informed consent. Synapse 3D software [40] is a program that automatically segments kidney images. According to the protocol of this software, we imported axial CT images into the program, after which the researcher directly marked the longest kidney length on the sagittal images reconstructed by the program. Segmentation was then executed. Subsequently, inaccurately segmented images were amended through consultations with two clinicians for each slice, and an accurately segmented image was obtained (Fig. 1).

The kidney volume determined by stereology was then obtained, along with the value measured by the ellipsoid method provided by the program. Obtaining the segmented kidney volume with satisfactory quality was completed within 1 s, but the overall process took about 30 min to 1 h per case because manual correction was performed for accurate segmentation (Supplementary Fig. 1). The supplementary Table 1 presents the statistical results of a comparison between automated stereology alone and manual correction with standard-dose CT (SDCT) or low-dose CT (LDCT). The differences of corrected – automatic volume were − 1.05(-18.6, 12.1) in SDCT and − 4.6(-28.1, 18.9) in LDCT.

### Statistical analysis

Descriptive statistics are shown as median (interquartile range (IQR)). The volume measurements from both sets of images were compared using R^2^. To determine the level of volume agreement between standard-dose CT and low-dose CT, Bland-Altman plots were constructed, and coefficient of variation, intra-class correlation coefficients (ICCs) were calculated. The data were analyzed with SPSS version 23.0 (IBM Corp., Armonk, NY, USA). All reported P-values are two-tailed, and the statistical significance threshold was set at P < 0.05.

## Results

The demographic characteristics of the 24 ADPKD patients included in this study are shown in Table 2. The median (IQR) age of patients was 47.1 (32.0, 78.5) years, and 11 patients (48.5%) were men. The median (IQR) eGFR was 86.7 (35.1, 114.1) mL/min/1.73m^2^. The median (IQR) creatinine level was 1.0 (0.8, 1.7) mg/dL and the median (IQR) cystatin C level was 0.9 (0.7, 2.2) mg/dL. Although most patients had a large kidney volume, their eGFR was still preserved. For standard-dose CT, the median (IQR) volume of the kidneys by stereology was 1492 (738, 2012) mL, whereas that obtained for low-dose CT was 1485 (714, 1948) mL. The absolute difference (standard – low) and the % difference ((standard – low)/standard x 100) of volume, mL were 22.6 (0.66, 81.81) and 2.67(-0.60, 4.21). The coefficient of variation and ICC were 0.02(0.01, 0.03) and 0.998, respectively (Table 2).

**Table 2:** **Demographic and clinical characteristics of 24 ADPKD patients**

n = 24	Standard dose	Low dose
Age, yr, median (IQR)	47.1 (32.0, 78.5)	
Male, n (%)	11 (45.8%)	
eGFR Cr-Cys, mL/min/1.73m^2^ (IQR)	86.7 (35.1, 114.1)	
Cr, mg/dL, median (IQR)	1.0 (0.8, 1.7)	
Cystatin C, mg/dL, median (IQR)	0.9 (0.7, 2.2)	
Volume by stereology, mL, median (IQR)	1492 (738, 2012)	1485 (714, 1948)
Absolute difference (standard – low) of volume, mL, median (IQR)	22.6 (0.66, 81.81)	
% Difference ((standard – low)/standard) of volume, mL, median (IQR)	2.67(-0.60, 4.21)	
Coefficient of variation, mL, median (IQR)	0.02(0.01, 0.03)	
ICC	0.998	
Volume by ellipsoid, mL, median (IQR)	1436 (687, 2195)	1364 (670, 1861)
DLP, mGy·cm, median (IQR)	219 (185, 265)	48 (37, 66)

*Abbreviations:* ADPKD, autosomal dominant polycystic kidney disease; IQR, interquartile range; eGFR, estimated glomerular filtration rate; Cr, creatinine; DLP, dose-length product

Additionally, the median (IQR) volume obtained using the ellipsoid technique was 1436 (687, 2195) mL with standard-dose CT and 1364 (670, 1861) mL with low-dose CT. The median (IQR) dose-length product (DLP) (a parameter that measures the amount of radiation exposure) was dramatically different, as predicted—namely, 219 (185, 265) mGy·cm for standard-dose CT and 48 (37, 66) mGy·cm for low-dose CT. The low-dose CT scans administered only 22% of the radiation dose of standard-dose CT (Table 2).

**Fig. 2:**
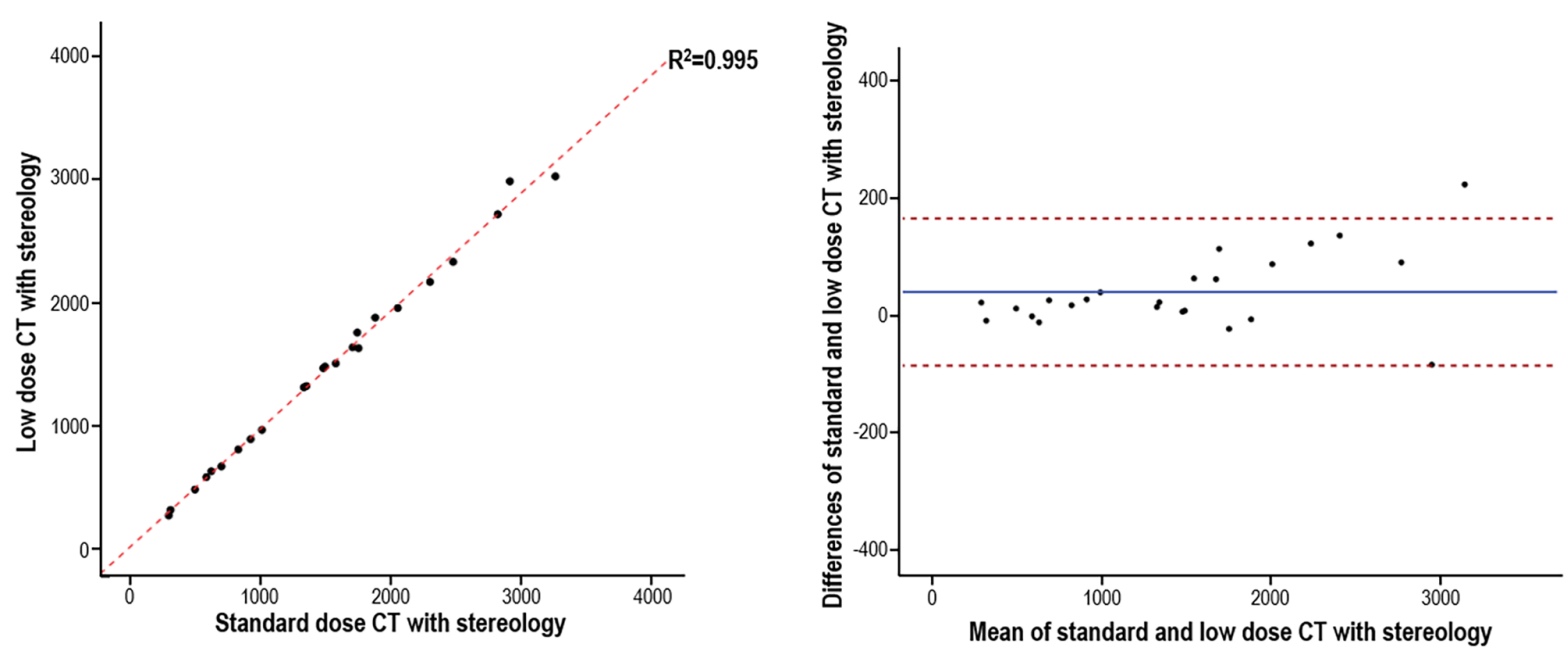
The correlation and agreement of standard-dose and low-dose CT volumetry. (Left panel) Linear regression analysis was done between standard-dose CT with stereology and low-dose CT with stereology. The value of R^2^ (coefficient of determination) was 0.995. (Right panel) Bland-Altman analysis was conducted to show the agreement of standard-dose and low-dose CT with stereology. CT, computed tomography

Based on the measurement outcomes of TKV, linear regression and Bland-Altman plots were applied to visualize the correlations and agreement of outcomes from low-dose CT and standard-dose CT with stereology. In linear regression analysis, the value of R^2^ was 0.995. The ICC was 0.998 using a two-way mixed-effects model where the clinician effect was random and the measurement effect was fixed. The Bland-Altman plot also presented fully consistent measurement outcomes for low-dose CT and standard-dose CT, except for one outlier where the TKV was over 3000 mL. These results mean that stereology using low-dose CT scans could be as valid as standard-dose CT (Fig. 2).

**Fig. 3:**
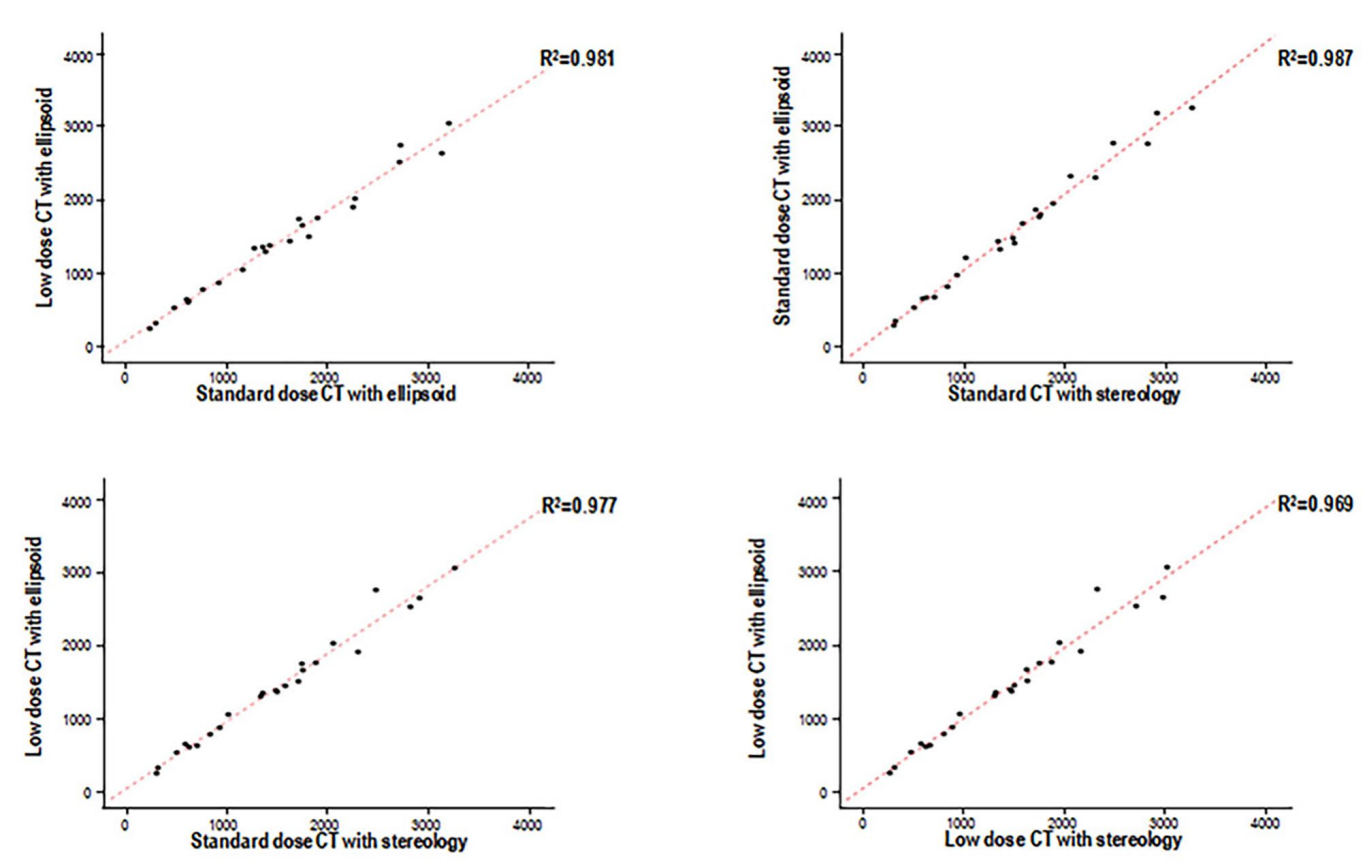
Correlation between the ellipsoid and stereology techniques with low-dose CT and standard-dose CT. Several graphs show the correlations of measurement outcomes through linear regression. Each graph presents the correlation between the ellipsoid and stereology techniques with low-dose CT and standard-dose CT, respectively. CT, computed tomography

Additionally, linear regression was performed and Bland-Altman plots were constructed to show whether the ellipsoid and stereology techniques were equally valid for low-dose CT and standard-dose CT (Fig. 3). The value of R^2^ between standard-dose CT and low-dose CT using the ellipsoid method was 0.981. The R^2^ values between the ellipsoid and stereology methods with standard-dose CT, between the ellipsoid and stereology methods with low-dose CT, and between the ellipsoid method with low-dose CT and stereology with standard-dose CT were 0.987, 0.969, and 0.977, respectively.

**Fig. 4:**
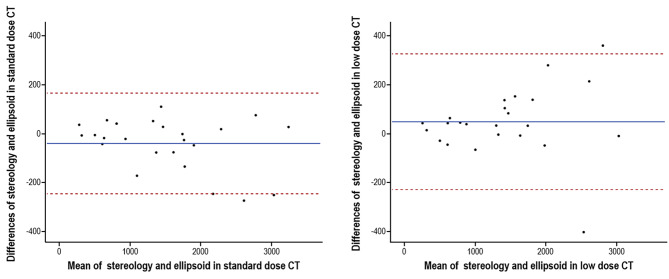
Agreement between the ellipsoid and stereology techniques with low-dose CT and standard-dose CT. Two graphs show the agreement of measurement outcomes through Bland-Altman analysis. Each graph presents the agreement between the ellipsoid and stereology techniques with standard-dose CT or low-dose CT, respectively

As shown in Fig. 2 (Right) and Fig. 4, in the Bland-Altman plots, except for some outliers, most of the TKV measurement outcomes were consistent with each other. Interestingly, all the outliers from the Bland-Altman plots, including stereology with low-dose CT and standard-dose CT (Fig. 2), occurred when the TKV was over 3000 mL. We also observed tendencies for an increasing difference between measurement outcomes between the ellipsoid and stereology techniques in low-dose CT and standard-dose CT as the TKV increased. This could lead to inaccuracy in TKV measurements as it increases, which implies the necessity of revising Synapse 3D software to achieve better TKV accuracy, especially when for ADPKD patients who are near the end stage.

## Discussion

Through linear regression analysis, the R^2^, ICC, and Bland-Altman plots, we found that TKV measurements of ADPKD patients using low-dose CT images with stereology were nearly equivalent to the results of the ellipsoid equation using standard-dose CT images or stereology volumetry using standard-dose CT. The correlations and agreement were all high enough to demonstrate the validity of TKV measurements by low-dose CT stereology, suggesting the possibility that low-dose CT-based stereology could substitute for standard-dose CT.

A limitation of this study is that it only included data from 24 patients. The sample size might have been insufficient to fully prove that TKV measurements through stereology using the Synapse 3D software can substitute for TKV measurements obtained using the ellipsoid technique or measurements based on standard-dose CT images, but the number of patients included in the study is not so small to undermine its validity. Additionally, although a previous study has already suggested the idea of using low-dose CT instead of standard-dose CT in TKV measurements [37], this study made a distinctive contribution by utilizing the Synapse 3D software, which now is widely used in medical imaging studies [38–41]. The former study found that results derived by volume measurement equations based on low-dose CT were valid as MRI-based planimetry using volumetry. Nonetheless, this study aimed to show that automated stereology-based volume measurements obtained using the Synapse 3D software could result in almost the same values between standard-dose CT and low-dose CT with a four-fold lower dose. The stereology-based TKV measurements obtained using low-dose CT images were not substantially different from those made by disciplined clinicians, meaning that many hospitals can now easily obtain accurate and prompt TKV measurements via the Synapse 3D software, a widely utilized program. Overcoming the inaccuracy of equations using kidney volumetry, an automated and elaborate process through 3D analysis would dramatically reduce the time consumed by making measurements for ADPKD patients.

Although the ellipsoid method is useful for measuring kidney volume quickly with a certain degree of accuracy, the results of this study imply that the ellipsoid technique might be an inefficient tool for the follow-up ADPKD patients with relatively small kidney volumes. As shown in Fig. 4, with the ellipsoid method, it should be considered that there may be a difference of TKV about 200–300 mL, and when low-dose CT with the ellipsoid method is used, the difference becomes larger. This discrepancy between these situations using standard dose CT and low-dose CT might further confirm the inaccuracy of the ellipsoid technique [25]. The average kidney volumes of adults are 146 mL in the left kidney and 134 mL in the right kidney [42]. As mentioned above, the Mayo classification considers patients with annual kidney size increments of 3-6% as belonging to class C-E and needing tolvaptan treatment. In this case, if the initial volume measurement in an ADPKD patient is small, an error of 200–300 mL in kidney volume would make it impossible to evaluate and classify patients with the ellipsoid equation considering the normal renal volume. For example, a patient in Mayo class C with an htTKV of 1500 mL might have an htTKV increase of more than 75 mL after 1 year, but the ellipsoid method would not be able to conclusively determine whether actual volume progression has occurred due to the error of 200–300 mL.

## Conclusions

In conclusion, low-dose CT showed comparable TKV measurement results to standard-dose CT in ADPKD patients using stereology in the Synapse 3D program. Therefore, low-dose CT might be used to minimize radiation exposure in ADPKD volumetry instead of standard-dose CT. We examined the correlation and agreement between stereology in standard-dose CT and low-dose CT and found high values. However, as shown in the Bland-Altman plots, there could be a significant error in volume measurements obtained using the ellipsoid technique, so it is recommended to use 3D stereology volumetry rather than the ellipsoid technique for the follow-up of ADPDK patients receiving tolvaptan.

### Electronic supplementary material

Below is the link to the electronic supplementary material.

Supplementary Material 1

## Data Availability

The datasets used and/or analyzed during the current study are available from the corresponding author upon reasonable request.

## List of Abbreviations

ADPKD: Autosomal dominant polycystic kidney disease
CT: Computed tomography
ICCs: Intra-class correlation coefficients
ESRD: End-stage renal disease
MRI: Magnetic resonance imaging
TKV: Total kidney volume
htTKV: Height-adjusted TKV
