# Supplementary material for: Non-contrast low-dose CT can be used for volumetry of ADPKD
Source: BMC Nephrol. 2023 Oct 26;24:317. doi: 10.1186/s12882-023-03359-z (PMC10604523; doi:10.1186/s12882-023-03359-z)
Supplement: Supplementary file 1 — Supplementary Material 1 [file 12882_2023_3359_MOESM1_ESM.docx]

Supplementary Figure 1.

A) Image showing automatic segmentation using Synapse software in standard dose computed tomography (CT). B) The segmentation of A) was manually adjusted, and the changed spots are shown by red arrows. C) Image showing automatic segmentation using Synapse software in low dose CT. D) The segmentation of C) was manually adjusted, and the changed spots are shown by red arrows.


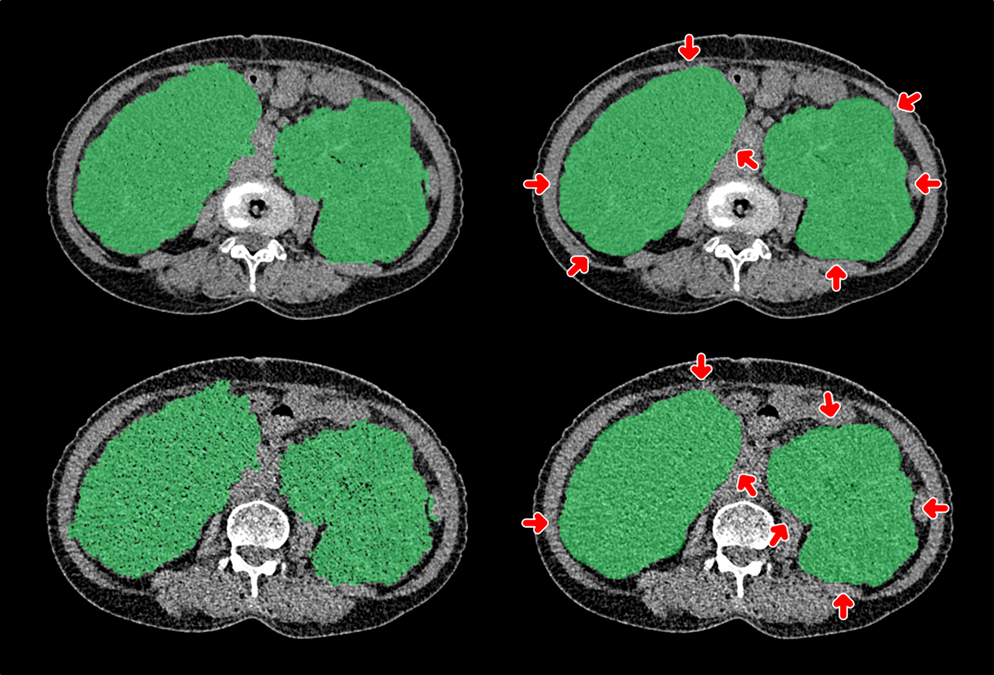


**A)**

**B)**

**C)**

**D)**

Supplementary Table 1.

| Differences in kidney volume (corrected - automatic) | Standard dose | Low dose |
| --- | --- | --- |
| Median (IQR) | -1.05(-18.6, 12.1) | -4.6(-28.1, 18.9) |
